# Duroline

**Published:** 1895-08-10

**Authors:** 


### EDITOR'S LETTER-BOX.

[Our correspondents are reminded that proxility is a great bar to publication, and that brevity of style and conciseness of statement greatly facilitate early insertion.]

### "DUROLINE."

Mr. George Walton (the secretary) writes, under date July 31st, 1895; in The Hospital of the 27th inst., in a paragraph setting forth the advantages of the material called duroline, manufactured by the company of which I am secretary. For this notice my directors tender you their thanks, but they consider that the statement that the material is highly inflammable is calculated to do the company much harm. The material is inflammable without doubt, but certainly not highly inflammable, as flame may be applied to the surface for some time without setting it on fire. If the match be applied to the edge it will soon burn, but without much flame, the wire gauze, which is in fact the basis of the material, preventing rapid or much burning. By the request of my directors I enclose a small sample of duroline that you may have an opportunity of verifying my statement. They would draw your attention to the fac that the insurance companies do not charge higher rates for the insurance of buildings where duroline is substituted for glass, and that a preference for this material as against glass has been claimed by more than one official connected with insurance companies as being much safer in several respects.

